# Assessment of the risk and characterization of non-melanoma skin cancer in Kindler syndrome: study of a series of 91 patients

**DOI:** 10.1186/s13023-019-1158-6

**Published:** 2019-07-24

**Authors:** Sara Guerrero-Aspizua, Claudio J. Conti, Maria Jose Escamez, Daniele Castiglia, Giovanna Zambruno, Leila Youssefian, Hassan Vahidnezhad, Luis Requena, Peter Itin, Gianluca Tadini, Ivelina Yordanova, Ludovic Martin, Jouni Uitto, Cristina Has, Marcela Del Rio

**Affiliations:** 1 Department of Bioengineering, Universidad Carlos III de Madrid, Leganés, Madrid, Spain; 2 Hospital Fundación Jiménez Díaz e Instituto de Investigación FJD, Madrid, Spain; 3 Epithelial Biomedicine Division, CIEMAT, Madrid, Spain; 4 Centre for Biomedical Network Research on Rare Diseases (CIBERER), U714 Madrid, Spain; 5 Laboratory of Molecular and Cell Biology, Istituto Dermopatico dell’Immacolata (IDI)-IRCCS, Rome, Italy; 6 Genetic and Rare Diseases Research Area, Bambino Gesù Children’s Hospital, IRCCS, Piazza Sant’Onofrio, 4, 00165 Rome, Italy; 7 Department of Medical Genetics, Tehran University of Medical Sciences, Tehran, Iran; 8 Department of Dermatology and Cutaneous Biology, Sidney Kimmel Medical College, Thomas Jefferson University, Philadelphia, PA USA; 9 Biotechnology Research Center, Department of Molecular Medicine, Pasteur Institute of Iran, Tehran, Iran; 10 Department of Dermatology, University Hospital Basel, Basel, Switzerland; 11 Pediatric Dermatology, Department of Physiopathology and Transplantation, Fondazione IRCCS Ca’ Granda, Ospedale Maggiore Policlinico di Milano, University of Milan, Milan, Italy; 12 Department of Dermatology and Venerology, Medical University Pleven, Pleven, Bulgaria; 13 Department of Dermatology, Angers University Hospital, Angers, France; 14 Department of Dermatology, University Medical Center Freiburg, Freiburg, Germany

**Keywords:** Kindler syndrome, SCC, Skin cancer, Bullous disease, Prevalence

## Abstract

**Background:**

Kindler Syndrome (KS) is a rare genodermatosis characterized by skin fragility, skin atrophy, premature aging and poikiloderma. It is caused by mutations in the FERMT1 gene, which encodes kindlin-1, a protein involved in integrin signalling and the formation of focal adhesions. Several reports have shown the presence of non-melanoma skin cancers in KS patients but a systematic study evaluating the risk of these tumors at different ages and their potential outcome has not yet been published. We have here addressed this condition in a retrospective study of 91 adult KS patients, characterizing frequency, metastatic potential and body distribution of squamous cell carcinoma (SCC) in these patients. SCC developed in 13 of the 91 patients.

**Results:**

The youngest case arose in a 29-year-old patient; however, the cumulative risk of SCC increased to 66.7% in patients over 60 years of age. The highly aggressive nature of SCCs in KS was confirmed showing that 53.8% of the patients bearing SCCs develop metastatic disease. Our data also showed there are no specific mutations that correlate directly with the development of SCC; however, the mutational distribution along the gene appears to be different in patients bearing SCC from SCC-free patients. The body distribution of the tumor appearance was also unique and different from other bullous diseases, being concentrated in the hands and around the oral cavity, which are areas of high inflammation in this disease.

**Conclusions:**

This study characterizes SCCs in the largest series of KS patients reported so far, showing the high frequency and aggressiveness of these tumors. It also describes their particular body distribution and their relationship with mutations in the FERMT-1 gene. These data reinforce the need for close monitoring of premalignant or malignant lesions in KS patients.

## Background

Kindler Syndrome (KS) is a rare autosomal recessive genodermatosis, considered a subtype of epidermolysis bullosa (EB), characterized by skin fragility with photosensitivity and acral blisters formation in young patients [1]. As they age, they develop progressive skin atrophy, premature aging, poikiloderma, palmoplantar hyperkeratosis, and pseudosyndactyly [2]. Mucosal manifestations are also common and include haemorrhagic mucositis and gingivitis, periodontal disease, premature loss of teeth, and labial leukokeratosis [3]. Interestingly, unlike other skin bullous diseases, KS is characterized by sensitivity to UV and dysregulation of oxidative stress [4], [5], [6].

KS is caused by mutations in the FERMT1, a gene that codes for kindlin-1, a protein associated to integrin and focal adhesions [7], [8].

Reports in the literature have documented the presence of SCCs in KS patients suggesting a predisposition of these patients to this neoplasia [9], [10]. However, a systematic study of cancer frequency and aggressiveness in a series of KS patients has not been carried out. Therefore, the relative risk of SCC at different ages cannot be predicted from the current published data on KS. Furthermore, although SCCs in KS have been described in individual case reports, published studies do no provide common behaviours, body location and the existence or lack of mutations in FERMT1 that can be directly associated with cancer in KS.

We have previously reported the largest series of KS patients from different countries and with the associated FERMT 1 (KIND1) genotype in patients of different ethnicities. The development of cutaneous cancer was noted in some patients; however, tumor characteristics and follow-up data were not provided [2].

In this paper we present a series of 91 cases, 69 cases previously published [2], [11] and 22 new cases unpublished, with the primary goal of establishing the incidence of SCC in KS patients, at different ages. We have also used this series, in some cases supplemented with data from the literature, to investigate other characteristics of SCCs in KS, such as tumoral prognosis and outcome, body distribution of the tumors and the existence or lack of KS mutations associated to SCC appearance. To our knowledge, this represents the first study published that characterizes as a whole, SCC in Kindler syndrome.

## Results

### Patients and development of SCC

Information about our patients including gender, age, genetic data and the presence of muco-cutaneous SCC is provided (Table 1).

**Table 1:** Characterization of the SCCs developed in the KS adult patients of this study. A total series of 91 KS patients is shown, 69 of them previously published and 22 new unpublished cased. SCC: Squamous Cell Carcinoma; WD: Well differentiated; AS: Altered splicing; RT: Reduced transcription. UD: Unpublished data; **: FERMT1 mutation confirmed (manuscript in preparation)

Pat #	Gender & Age	Mutation DNA nucleotide change (cDNA)	Mutation (gDNA) in Chromosome 20	Mutation (gDNA) in gene FERMT1	Protein	Epithelial Cancer/ Histopathology	Cancer onset (Age)	Outcome	Refs
1.	M, 15	c.1718 + 2 T > C	g.6084038A > G	35516	AS	–			[2]
2.	F, 16	c.190G > T	g.6116006C > A	3548	p.E64X	–			UD
3.	M, 17	c.614G > A	g.6110430C > T	9124	p.W205X	–			[7]
4.	M, 17	c.328C > T	g.6115868G > A	3686	p.R110X	–			[12]
5.	M, 18	c.1051G > T	g.6096940C > A	22614	p.E351X	–			[2]
6.	F, 18	c.676dupC	g.6110368dupG	9186	p.Q226PfsX17	–			[2]
7.	M, 18	c.910G > T	g.6097571C > A	21983	p.E304X	–			[2] and [13]
8.	M, 20	g.70250_74168del/ c.-20A > G g.6116239_ 6120157	g.6122775 T > C	− 3221	p.P381Rfs: PTC/RT	–			[14]
9.	F, 20	c.957 + 1G > A	g.6097523C > T	22031	AS: In intron 7 at donor splice site	–			[15]
10.	M, 22	c.1729delA	g.6079567delT	39987	p.S577AfsX14	–			[2]
11.	F, 23	c.994_995delCA	g.6096996-6096997delTG	22558	p.Q332fsX9	–			[15]
12.	M, 23	**	–	–	–	–			UD
13.	M, 24	c.1161delA	g.6089068delT	30486	p.A388LfsX14	–			[2]
14.	M, 24	c.1161delA	g.6089068delT	30486	p.A388LfsX14	–			[2]
15.	F, 24	c.676dupC	g.6110368dupG	9186	p.Q226PfsX17	–			[2] and [16]
16.	M, 24	c.1139 + 2 T > C / c.889A > G	g.6094937A > G/ g.6097592 T > C	21962	AS/ p.R297G	–			[15]
17.	F, 24	c.910G > T	g.6097571C > A	21983	p.E304X	–			[15]
18.	M, 25	g.-711-1241del g.6045219_6047230del	g.6088988-6110333del	30566	RT	–			[14] and [17]
19.	M, 25	c.170C > A	g.6116026G > T	3528	p.S57X	–			[2] and [16]
20.	F, 25	g.70250_74168del g.6116239_ 6120157del	g.6084280del	35274	AS Large deletion p.P381Rfs PTC	–			[2]
21.	M, 25	c.676C > T	g.6110368G > A	9186	p.Q226X	–			UD
22.	M, 26	c.1718 + 2 T > C	g.6084038A > G	35516	AS	–			[15] and [18]
23.	M, 26	c.550-551insA	g.6110493-6110494insT	9061	p.S184LfsX1	–			[15]
24.	F, 26	c.676C > T	g.6110368G > A	9186	p.Q226X	–			UD
25.	F, 27	g.70250_74168del g.6116239_ 6120157del	g.6084280del	35274	Large deletion p.P381Rfs PTC	–			[2]
26.	M, 27	c.1161delA	g.6089068delT	30486	p.A388LfsX14	–			[2]
27.	M, 27	c.910G > T	g.6097571C > A	21983	p.E304X	–			[15]
28.	F, 28	c.676C > T	g.6110368G > A	9186	p.Q226X	–			UD
29.	F, 29	c.1719-1G > A	g.6079578C > T	39976	AS	–			[2]
30.	F, 29	c.1139 + 740G > A (IVS9 + 740G > A)	g.6094199C > T	25355	pseudoexon	–			[19]
31.	F, 29	c.889A > G/ c.1139 + 2 T > C	g.6097592 T > C/ g.6094937A > G	21962	AS/ p.R297G	–			[15]
32.	M, 29	c.1718 + 2 T > C	g.6084038A > G	35516	AS	–			[15] and [18]
33.	F, 29	c.550-551insA	g.6110493-6110494insT	9061	p.S184LfsX1	–			[15]
34.	M, 29	c.1383C > A	g.6085276G > T	34278	p.Y461X	–			[15]
35.	F, 29	c.676C > T	g.6110368G > A	9186	p.Q226X	–			UD
36.	F, 30	c.676C > T	g.6110368G > A	9186	p.Q226X	–			UD
37.	F, 31	c.676C > T	g.6110368G > A	9186	p.Q226X	2 SCC: WD& early invasive (dorsal hand, between 4th and 5th fingers)	29	Alive	UD
38.	M, 31	c.550-551insA	g.6110493-6110494insT	9061	p.S184LfsX1	–			[15]
39.	M, 31	c.676C > T	g.6110368G > A	9186	p.Q226X	–			UD
40.	M, 31	c.676C > T	g.6110368G > A	9186	p.Q226X	–			UD
41.	F, 31	**	–	–	–	–			UD
42.	F, 32	c.1714_1715insA	g.6084043-6084044insT	35511	p.V572DfsX4	–			[2]
43.	M, 32	c.550-551insA	g.6110493-6110494insT	9061	p.S184LfsX1	–			[15]
44.	F, 32	c.1383C > A	g.6085276G > T	34278	p.Y461X	–			[15]
45.	F, 32	c.676C > T	g.6110368G > A	9186	p.Q226X	–			UD
46.	M, 32	c.676C > T	g.6110368G > A	9186	p.Q226X	–			UD
47.	F, 33	c.889A > G/ c.1139 + 2 T > C	g.6097592 T > C/ g.6094937A > G	21962	AS/ p.R297G	–			[15]
48.	M, 33	c.328C > T	g.6115868G > A	3686	p.R110X	–			[12]
49.	M, 34	c.958-1G > A	g.6097034C > T	22520	AS	–			[2]
50.	M, 34	c.456dupA	g.6112553dupT	7001	p.D153RfsX4	SCC: WD, early infiltrating, Oral mucosa, extending to tongue, lymph node metastases	33	Deceased at 35 years	[2]
51.	M, 34	c.1718 + 1G > A	g.6084039C > T	35515	AS	–			[2]
52.	M, 34	c.1383C > A	g.6085276G > T	34278	p.Y461X	–			[15]
53.	M, 34	c.676C > T	g.6110368G > A	9186	p.Q226X	–			UD
54.	F, 36	c.1209C > G	g.6089020G > C	30534	p.Y403X	–	Breast cancer	39 years	[2] and [20]
55.	M, 36	c.1176 T > G	g.6089053A > C	30501	p.Y392X	–			[15]
56.	M, 36	c.550-551insA	g.6110493-6110494insT	9061	p.S184LfsX1	–			[15]
57.	M, 36	**	–	–	–	–			UD
58.	M, 37	c.1365–1371 + 3del	g.6087774-6087783del	31780	AS exon11	–			[2] and [21]
59.	F, 37	c.550-551insA	g.6110493-6110494insT	9061	p.S184LfsX1	–			[15]
60.	M, 37	c.676C > T	g.6110368G > A	9186	p.Q226X	–			UD
61.	M, 39	g.6109607-6112272del	g.6109607-6112272del	9947	Deletion of exon4	–			[15]
62.	F, 40	c.550-551insA	g.6110493-6110494insT	9061	p.S184LfsX1	2 Metastasizing SCC to lymph nodes Fingers and hand	38	Deceased at 40 years	[15]
63.	M, 40	c.889A > G / c.1139 + 2 T > C	g.6097592 T > C/ g.6094937A > G	21962	AS/ p.R297G	–			[15]
64.	M, 40	c.676C > T	g.6110368G > A	9186	p.Q226X	–			UD
65.	F, 40	c.676C > T	g.6110368G > A	9186	p.Q226X	–			UD
66.	M, 42	c.373delT	g.6115823delA	3731	p.C125AfsX4	–			[2]
67.	M, 42	c.1179G > A	g.6089050C > T	30504	p.W393X	–			UD
68.	F, 43	g.6000982_6009222del (IVS13_15del)	g.6076879_6084040del	42675	PTC (exon14)	–			[2]
69.	F, 45	g.70250_74168del g.6116239_ 6120157del	g.6084280del	35274	p.P381Rfs Large deletion	–			[2]
70.	M, 47	g.6000982_6009222del (IVS13_15del)	g.6076879_6084040del	42675	PTC (exon14)	–			[2]
71.	M, 47	c.889A > G / c.1139 + 2 T > C	g.6097592 T > C/ g.6094937A > G	21962	AS/ p.R297G	–			[15]
72.	M, 47	**	–	–	–	–			UD
73.	M, 48	c.958-1G > A	g.6097034C > T	22520	AS	2 Infiltrating SCC: WD Upper Lip and dorsum of the hand	43	Alive	[2] [22]
74.	M, 49	c.676dupC	g.6110368dupG	9186	p.Q226PfsX16	–			UD
75.	M, 50	c.910G > T	g.6097571C > A	21983	p.E304X	Metastasizing and poorly differentiated SCC on the foot. Diffuse metastases (skin & lung)	48 years	Deceased at 50 years	[2]
76.	M, 50	c.958-1G > A	g.6097034C > T	22520	AS	–			[2]
77.	M, 51	c.889A > G / c.1139 + 2 T > C	g.6097592 T > C/ g.6094937A > G	21962	AS/ p.R297G	–			[15]
78.	M, 52	c.328C > T	g.6115868G > A	3686	p.R110X	Metastasizing SCC to lymph nodes (Lip)	48	Deceased at 52 years	[15]
79.	F, 60	g.6000982_6009222del (IVS13_15del)	g.6076879_6084040del	42675	Large deletion and PTC in exon14 PTC (exon14)	2 SCC: WD & early invasive Lower lip and dorsum of the hand	45, 53	Deceased at 60 years	[2], [11] and [23]
80.	F, 56	c.152-2delAGinsCT	g.6116046delinsAG	3508	AS	–			[2]
81.	M, 56	c.299-301del	g.6115895-6115897del	3659	p.R100del	–			[2] and [24]
82.	F, 58	c.96-97delAG	g.6119458-6119459delCT	96	p.R32SfsX63	Poorly differentiated SCC. Left arm	52	Amputation of left arm	[2]
83.	M, 65	c.1209C > G	g.6089020G > C	30534	p.Y403X	1 SCC: WD Invasive, left hand	57	Alive	[2]
84.	M, 59	c.889A > G / c.1139 + 2 T > C	g.6097592 T > C/ g.6094937A > G	21962	AS/ p.R297G	–			[15]
85.	M, 60	c.958-1G > A	g.6097034C > T	22520	AS Intron7	1–3 Infiltrating SCC, all WD; 4, 5 in situ SCC; 1 upper lip; 2 third right finger; 3 right hand dorsum; 4 lower lip; 5 fourth interdigital space of the left hand	43 (44, 45, 45, 56)	Alive	[2] and [22]
86.	M, 61	c.1139 + 740G > A (IVS9 + 740G > A)	g.6094199C > T	25355	Pseudoexon				[19]
87.	F, 63	c.1761 T > A	g.6079535A > T	40019	p.Y587X				[25]
88.	M, 65	g.70250_74168del	g.6084280del	35274	p. P381R *fsX36* *PTC* (large deletion: exons 10 and 11)	Infiltrating SCCs, all WD 1 lower gingival mucosa and regional lymph nodes; 2 scalp; 3 lower lip	52 (53, 55)	Alive	[2]
89.	M, 71	c.862C > T	g.6097619G > A	21935	p.R288X				UD
90.	M, 71	c.328C > T	g.6115868G > A	3686	p.R110X	Multiple in situ SCC: Hyperkeratotic SCC in situ. Face and hands.	Start at 50 years	Alive	[2]
91.	F, 79	c.328C > T	g.6115868G > A	3686	p.R110X	Invasive SCC, BCC, in situ SCC. Face and hands.	Start at 66 years	Alive	[2]

In our series of 91 patients, 13 developed non-melanoma skin tumors. Most of these patients presented multiple tumors, which resulted in a total of 25 tumors in the skin and 2 in the oral mucosa. With the exception of one BCC, all of the neoplastic lesions were SCC of variable histological grades but mostly well differentiated SCC.

When the patients were stratified by age, and the cumulative incidence calculated, our data showed that the earliest SCC appeared in a 29 year old woman and the cumulative risk of SCC increased with age to reach 66.7% in patients over 60 years of age.

**Fig. 1:**
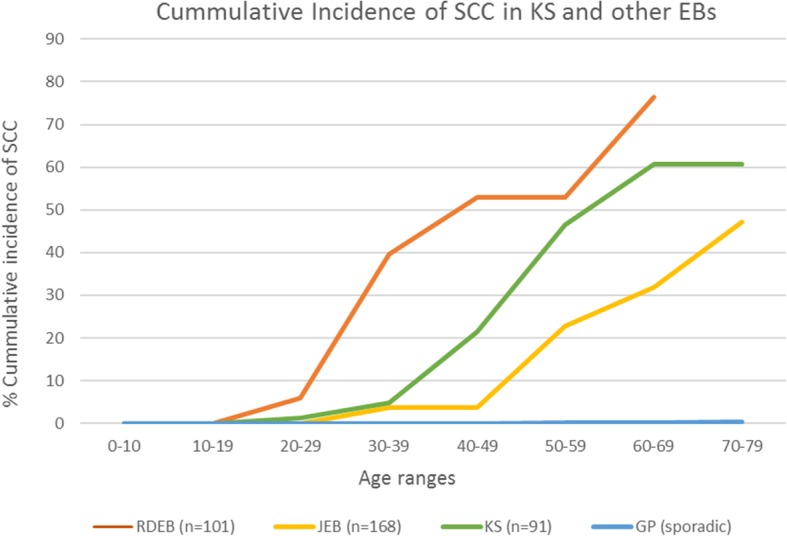
Calculated cumulative risk of the first squamous cell carcinoma in Kindler syndrome patients. For comparative purposes, we have also plotted the calculated cumulative risk described for RDEB, JEB and the general population which were taken from the literature [26], [27]

The SCC development cumulative risk in KS was compared to that reported for other types of EB patients and spontaneous SCC (Fig. 1). The KS profile is different from recessive dystrophic EB (RDEB) in which SCCs develop earlier and reach a higher cumulative risk and are also different from the generalized non-Herlitz types of Junctional EB (JEB) patients profile [28], [29], which showed a lower incidence and later onset. Fig. 1 shows the data from Fine et al., although this data has been somewhat controversial [29], [30]. Furthermore, in a series of one of our authors, the frequency appears much lower with only 1 SCC in 70 patients with Collagen XVII mutation and none in patients with Laminin322 or Integrin α6β4 mutations (Has, C. et al. unpublished results). In contrast, the incidence of sporadic SCC of sun exposed areas in the general population was much lower (0.001–0.005%), even in older patients (Fig. 1) [31], [26].

The outcome of SCCs (Table 1) in these patients has been variable leading to premature demise in five patients and arm amputation in another. Overall, 53.8% (7/13 cases) of the patients with SCC present metastasis .

Regarding mortality, 5/13 KS patients (38.5%), that developed SCC died as a direct consequence of the tumor, in a period that varied between 2 and 7 years, with an average of 40.8 months. This percentage(50%) is consistent with the previously reported data for KS-SCC [11], but much higher than the 5–10% of mortality described for sporadic tumors [31], [26]. Their detailed demographic, clinical and histopathological features are presented in Table 2.

**Table 2:** All the KS patients that have developed SCC, ordered by the age at which the first tumor appeared. Yellow patients have been published previously to this study. SCC: Squamous Cell Carcinoma; WD: Well differentiated; MD: Moderately differentiated; AS: Altered splicing; RT: Reduced transcription; PTC: Premature termination codon; FS: Frameshift; UD: Unpublished data

Gender, Age	Mutation	Protein	Age of cancer	Localisation of SCC	Histopath. of SCC	Outcome	Refs
M, 16	ISV9-6 T > A c.1140-6 T > A	FS and PTC	16	Right lower leg	WD aggressive SCC	Present for 10 years as a hyperkeratotic plaque, then progress in 2 months. Deceased.	[32]
F, 23	UD	UD	23	Right hand	Ulcerative and invasive MD SCC	Methastasic and Nodes affected	[33]
M, 40	c.1761 T > A and c.1089del/c.1089 + 1del	p.Y587X and Exon 8 skipped. (p.L363 fs)	27	Palm of the right hand	SCC recurrent 4 times	Recurrent SCC	[34]
F, 31	c.676 C > T	p.Q226X	29	Fingers and dorsal hand	WD early agressive	Still alive	UD
M, 34	c. 456dupA	p.D153R fsX4	33	Oral mucosa, extending to tongue, lymph node metastases	WD, early infiltrating SCC with metastases	Deceased at 35 years	[2]
F, 42	c.750G > A	p.W250X	34	Hard palate	WD SCC: unresectable treated with radiation	2 years follow up no recurrence. Now deceased.	[35]
F, 40	c. 550-551insA	p. S184 L fsX1	38	Fingers and hand	SCC metastasic to limph nodes	Deceased at 40 years	[15]
M, 49	c.910G > T	p. E304X	42, [20, 24]	Right shoulder, upper lip, dorsum of the right hand	SCC	UD	[36]
M, 60	c.676insC	p.W250X	43, 45	Lip and later in penis	SCC	UD	[37]
M, 48	c.958-1G > A	AS Intron 7	43	Upper Lip and dorsum of the hand	2 infiltrating WD SCC	Still alive	[2] and [22]
M, 60	c.958-1G > A	AS Intron 7	43 (44, 45, 45, 56)	1 upper lip; 2 third right finger; 3 right hand dorsum; 4 lower lip 5 fourth interdigital space of the left hand	1–3 Infiltrating SCC All WD; 4, 5 in situ SCC.	Still alive	[2]
F, 60	g.6000982_6009222del	IVS13_15del Large deletion and PTC in exon14	45, 53	1 SCC of the left hand 2 SCC of the lower lip	1 WD 2 WD early invasive	Deceased at the age of 60 years	[23] and [11]
M, 49	c.910G > T	p. E304X	48	Foot	Poorly differentiated SCC Metastasizing Diffuse metastases (skin & lung)	Deceased at the age of 50 years	[2]
M, 52	c.328C > T	p. R110X	48	lip	SCC metastasic to limph nodes	Deceased at the age of 52 years	[15]
M, 71	c.328C > T	p. R110X	50	Face and hands	Hyperkeratotic SCC in situ Multiple in situ SCCs	Still alive	[38]
F, 58	c.96_97delAG	p. R32S fsX63	52	Left arm	Poorly differentiated SCC	Amputation of left arm	[2]
F, 52	c.95_96delAG	p.R32fsX63	52	Lower lip	SCC	No follow up	[39]
M, 65	g.70250_74168del	p. P381R *fsX36*	52 (53, 55)	1 lower gingival mucosa and regional lymph nodes; 2 scalp; 3 lower lip	Infiltrating SCC, all WD	Still alive	[2]
F, 55	UD	UD	55	buccal mucosa extended to both lips	SCC	Treated with radiation therapy with partial response	[40]
M, 57	c.328 C > T	p. R110X	57	Left hand	moderate-to-poorly differentiated deeply invasive SCC	Recurrent, requiring axillary node clearance and amputation.	[10] and [41]
M, 65	c.1209C > G	p. Y403X	57	Left Hand	Invasive SCC WD	Still alive	[2]
F, 78	c.328C > T	p. R110X	66	Face and hands	Invasive SCC, BCC, In situ SCC	Still alive	[38]

The information in Table 2, includes grading of KS-SCC in 26/37 tumors (17/22 KS patients with histological data available). SCC considered as well-differentiated forms were the majority of the cases (22/26: 84.6%), moderately to poorly differentiated SCCs, were only presented in 4/26 (15.38%).

In order to study gender influence on the development of the KS-SCC, Table 3 was expanded to include previously published studies as well as the patients in this study. Our analysis did not reveal a significant difference in the development of SCC in male and female KS patients (Table 3).

**Table 3:** Gender and age distribution of SCCs appearance in all the KS patients that has been reported

Patients with carcinoma (including bibliography)			
Age range	Male	Female	Total
16–19	1	0	1
20–29	1	2	3
30–39	1	2	3
40–49	6	1	7
50–59	4	3	7
60–69	0	1	1
70–79	0	0	0
Total	13	9	22

Even though a difference in the total number of tumors in men and women cannot be observed, there seems to be a tendency in women to develop tumors at earlier ages (4/9: 44% before the 40’s), whereas in men, there was an abrupt increase after 40 years of age (10/13: 77%).

### Location of tumors

**Fig. 2:**
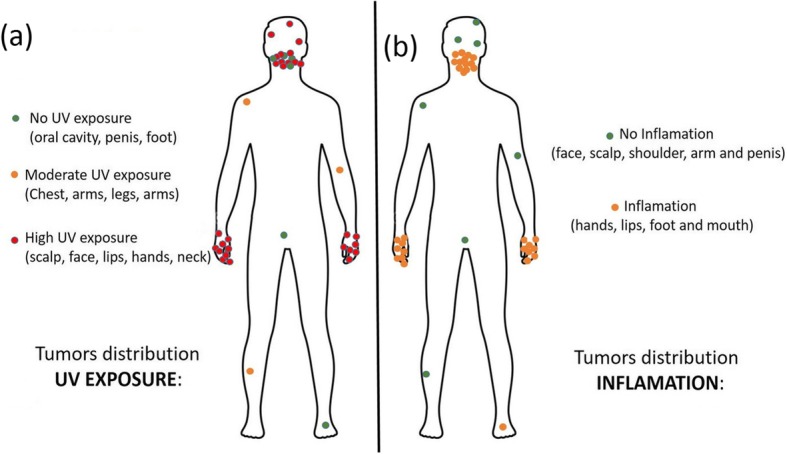
Representation of the body distribution of KS-SCCs. **a** The key color, classifies every tumor in different areas according to its´ UV exposure: Non-exposed (green), Moderately-exposed (orange) and Highly-exposed (red). **b** The key color, classifies every tumor in different areas according to whether the zone has been described as chronically inflamed in KS: no inflammation (green) and chronic inflammation (orange)

The location of the tumors in the body is shown (Fig. 2). All the KS-SCC described in the literature were also included in the figure [11], thus, a total of 37 SCC was used for this analysis. Furthermore, when tumors of this study and tumors from the literature were segregated, results were nearly identical (data not shown).

It can be observed that the majority of the tumors (28/37) developed in sun exposed areas, with the exception of 6 SCC of the oral mucosa, foot and penis, with 3 of them in moderately sun exposed areas (Fig. 2a). Surprisingly, most tumors were restricted to just two areas of the body; the face, particularly around the mouth (13/37 tumors), and hands (16/37 tumors); that together represented 78% of the KS-SCCs. Other sun-exposed areas were minimally affected, which resulted in a unique distribution, that overlaps with areas of chronic inflammation in KS patients [42], [43]. This pattern is different from sporadic [26] JEB [29] and RDEB associated SCCs [44].

The body distribution of SCC was represented with a key color. These classified every tumor if the zone was considered as a sun exposed area (Fig. 2a) and if the zone had been described as chronically inflamed in KS (Fig. 2b). It can be observed that, in KS-SCC there is a perfect coincidence between areas of chronic inflammation and tumor development.

### Mutations in SCC bearing KS patients

**Fig. 3:**
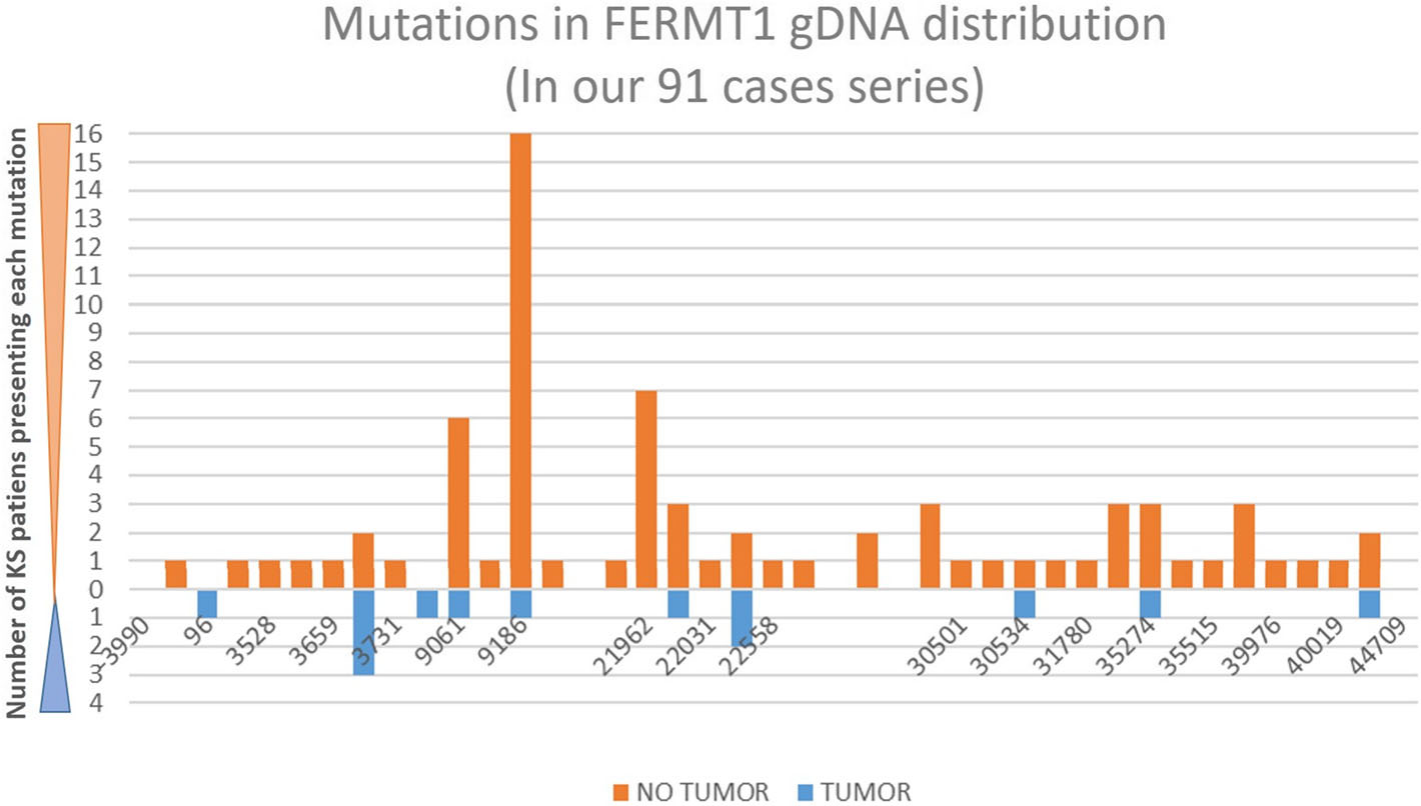
Distribution of the mutations in our patient series, along the gDNA of FERMT1. In the X axis, it is represented by the length of the FERMT1 gene, considering the + 1 position as the ATG. For that reason, the gene is from − 3990 to + 44709 positions. The Y-axis, shows the number of patients presenting with each mutation, in orange (free of SCC) and in blue (developed SCC). Four patients with no mutational information available were excluded from this figure

In order to discern if the predisposition to develop SCC was related with a pattern in the mutational spectra, gDNA mutations were represented (Fig. 3). In our series of KS patients, no hot spot, at genomic level, associated to SCC development was identified. Our data showed that SCC developed in patients with 10 different FERMT1 mutations. In most cases, there was one patient with SCC, for each mutation, with the exception of two mutations present in multiple patients. Three patients with the mutations c.328C > T (p.R110X; g.6115868G > A) developed a total of 5 tumors and 2 patients with the mutation c.958-1G > A (altered splicing in intron 7; g.6097034C > T) developed 7 tumors. Thus, these two mutations were characterized not only for the higher frequency of patients with SCC, but also for a higher multiplicity of tumors. Another interesting mutation was c.96-97delAG (p.R32SfsX63 g.6119458-6119459delCT) presented in a single SCC bearing KS patient, while all other mutations in patients with SCC were also presented in patients free of neoplasia. We have represented the mutations for all our 91 case series (only 87 with mutational information available) for those who are free of neoplasias and those that developed SCC (Fig. 3). When we compared the spectra of mutations of patient bearing tumors with the tumor free patients we found an interesting pattern.

From our data (Fig. 3 and Table 1), we can see that 10 out of 13 KS patients that developed SCCs, presented mutations that resulted in stop codons being located closer to the 5’end of FERMT1, which resulted in short transcripts that were likely to be degraded. The other three mutations were also frame shifts or alternative splicing which are likely to generate an unstable protein. In contrast, the spectra of mutations reported in the literature, in patients with and without tumors, showed a more even distribution all along the gene and with a higher representation at the sequence encoding carboxy terminus. In addition, according to the literature, non-sense mutations represent 37.5% of the total KS mutations [2] which is consistent with our study (36.6%). In patients with SCC, this proportion increases up to 60% (6 out of 10 reported mutations). Taking together all of this data, suggests than even though specific mutations do not have a predictive value in the development of SCC, there is a different profile of mutations in patients bearing tumors and tumor free patients.

## Discussion

In a case series of 91 KS patients, we have determined that KS patients have a high risk for developing SCC. The data from our case series indicated that SCCs appeared relatively early compared with sporadic SCC, but occurred about 10 to 20 years later than in RDEB, which is the type of EB with the highest risk of cancer [28]. On the other hand, SCC in JEB showed a higher latency and a lower incidence than KS.

The youngest patient that developed SCC in our series was a 29 year old female. It is worth noting that consistent with our study, there are only three younger KS patients of 16, 23 and 27 described with SCC in the literature [32], [33], [34]. It can be inferred that development of SCC in young patients is rare and in the case of paediatric patients is extremely rare or non-existent.

The cumulative incidence of SCC in KS increases after the early onset at 29 years old, to reach a maximum at 60 years old with a cumulative risk of 67%.

Of note, the body localization of cutaneous cancers in KS is also unique. Unlike RDEB and JEB, in which, the preponderance of lesions in the lower extremities is clear, KS tumors arose mainly in the mouth and hands. Interestingly, KS has been shown to have an oversensitivity to UV, but other areas of the body, highly exposed to the sun, were minimally affected [45]. This particular distribution was confirmed including 9 other KS patients that developed SCCs which were taken from the literature. Tumors in non-exposed areas were less common and were restricted to 6 patients; 4 in the oral mucosa, one, in the penis and another in the foot. RDEB and JEB SCCs are associated with chronically non-healing ulcerations, while in KS, the cutaneous condition improves with age. The adult skin in KS is characterized by atrophy, poikiloderma as well as inflammation of the mouth, hands and feet [42], [41]. These differential distributions support the notion that distinct pathogenic mechanisms may underlie cancer development in KS as compared to the other major EB types. Photosensitivity is a unique feature of KS among the EB disease spectrum, and loss of kindlin-1 is associated with an up-regulation of pro-inflammatory cytokines in keratinocytes, at least in part mediated by UV exposure with subsequently impaired DNA repair [4]. Furthermore, in KS we have observed mitochondrial damage with an increase in oxidative stress [5]. KS has also been characterized by strong inflammation in the mouth and hands [42], [43], [41]. Thus, SCC seems to develop by a synergy of UV exposure together with the stimulus of inflammation, in the mouth and hands, which results in the peculiar distribution of these tumors.

Interestingly, as in other EB related cancers, the SCC of the KS patients has the potential to be highly aggressive [28] leading to amputation [32], [33], [10] and early demise [32]. In agreement, in our series, five patients died as a consequence of the tumors, and another patient showed local metastasis which resulted in an amputation of an arm.

The mechanisms involved in the aggressive nature and early onset of SCC associated with ulcerative disease and chronic inflammation have not been completely elucidated. The putative role of Kindlin-1 has been widely discussed by Rognoni et al. (2014) which focused on the activation of TGF-β–mediated growth-inhibitory signals in the KS mouse model [46]. Moreover, TFG-beta has been described as a crucial factor in other bullous diseases modifying disease severity in RDEB, through the promotion/inhibition of a fibrotic matrix [47]. More recently, our group has underscored the role of the fibroblasts of KS, RDEB, and XPC, in the pathogenesis of these diseases, observing a higher activation of the TGF-β signalling pathway [48]. Taking together all these data and consistent with the body distribution described in this study, a potential mechanistic role of the TGF-β in the aggressive nature of KS-SCC becomes even more important, and should be further characterized.

In the last few years, a role of the stroma has been considered determinant and at least partially responsible for the early onset and aggressiveness of these tumors [48], [49], [50]. More recently; however, careful genetic studies by Cho et al. (2018) have shown that driver mutations seem to be shared between RDEB and sporadic skin tumors in sun exposed areas and other highly aggressive SCC, but unlike the sporadic tumor genes, RDEB showed a profile of endogenous mutations associated with APOBEC [51]. The alteration of these genes seems to be related with inflammation and is probably responsible of the differences between sporadic tumors and tumors associated with chronic ulcerative diseases and inflammation. Based on this data, we speculate that similar mechanism may occur in KS, in which tumors appear in areas that are both exposed to UV and present higher inflammation (i.e. mouth and hands).

In regard to the nature of FERMT1 mutations in patients that developed SCC, we showed that there are no clear hot spots or even predominant mutations in our cohort. Only two mutations are found in two and three patients, respectively. Although some mutations appear in this study more frequently associated with SCC (96, 3731, 22558 and 30534), there is not enough statistical significance to consider that they have a predictive value. In addition, none of the mutations seems to be carcinogenic ¨per se¨ since several mutations are found in patients bearing cancer and also in patients in which SCC have not been detected. However, the mutational distribution in FERMT1 and the type of mutation in the KS patients with SCC, was clearly different from that of the patients that did not develop SCC. While mutations in the overall population are distributed along the whole length of the gene, the mutations in the patients bearing SCC are mostly present in the N terminal part of the gene. Furthermore, most of them result in stop codons, which lead to short mRNA that are likely to be degraded. Taken together, these data suggest that most of the patients were null for kindlin-1, which is a paradoxical result, taking into account that there is a reasonable body of literature suggesting a pro-carcinogenic role of this protein. Overexpression of kindlin 1 has been reported in breast, lung, colon and esophageal cancers [52]. Furthermore, overexpression of this protein has also been associated with a poor prognosis in osteosarcoma [53] as well as breast and lung carcinomas and pancreatic cancer [54]. Based on these results, we conclude that kindlin1 may have a pro-carcinogenic or anticarcinogenic potential depending on the context as has been shown in other genes such as E2F, which can act as a tumor suppressor gene or an oncogene depending on the context [55].

A limitation of the present study is the relatively low number of cases, particularly in older patients. However, since the total population of reported patients with KS is less than 300, the report of 91 patients can be considered as a representative sample for such a rare disease. Similarly, in ideal circumstances, the risk of a disease should be assessed in a more homogeneous population. Our series included people from different countries and ethnicities. However, given the low prevalence of this disease, it would be impossible to do a study of these characteristics in a homogeneous population. Furthermore, our study analyzed 26 carcinomas in 13 different patients being so far 19, the total number of KS-SCC previously reported in the literature [11].

## Conclusions

Overall, this study predicts that more than half of patients with KS will develop SCC in their lifetime and among them, 53.8% of patients will develop metastatic disease with a high possibility of a lethal outcome. Thus, it is important to stress the need for close monitoring of these patients, aiming at early SCC detection (at pre-malignant stages and/or initial developmental stages) to avoid progression of the tumor, particularly in older patients when the symptoms of the disease are less severe and therefore, the monitoring may be less strict.

## Methods

### Patients and tumors

Patients for this retrospective study were recruited from different institutions in Europe and the United States. The core of our series are 34 patients previously described by Has et al. [2] to which, we included 57 new patients who were diagnosed with KS by clinical and molecular methods and who had records of tumor development as well as their characteristics [19]; [14]; [15]. All the patients included in this study were older than 16 years, the age of the youngest KS patient who developed a SCC [32]. In four patients from our series, FERMT1 mutations were not available and therefore, these patients were not included in the mutational distribution study (Fig. 3).

This series of 91 patients was used primarily to determine the incidence of SCC in KS, which has not been previously reported. In order to investigate other characteristics of KS-SCC, we have supplemented the information of our series with data from the literature.

For the SCC body distribution study, we have used 37 tumors, coming from 13 patients of our series and 9 additional KS patients from the literature [11]. To our knowledge, all the tumors and information available reported until now, are included here. Information regarding pedigree was not available for this study. To summarize, different groups of patients have been included in each analysis depending on the information available from them (mutations, follow up, histopathological characterization of the tumors,…). For cumulative risk calculation, 91 KS patients have been included (13 of which developed SCC) as was mentioned before and are shown in Table 1. For mutation distribution analysis (Fig. 3), only 87 KS patients were included from our 91 patients series, because 4 patients have no mutational information available (Patients #12, 41, 57 and 72 from Table 1). The SCC body distribution analysis shown in Fig. 2 was analyzed in 22 KS patients (from the bibliography and from our series) who developed a total of 37 tumors. The same group of patients was used to elaborate Table 2 that describes all the SCC bearing patients, but only 26/37 tumors had complete histopathological data available. For gender and age distribution analysis (Table 3), the same group of 22 SCC-KS patients was studied.

### Distribution of mutations along the FERMT1 gene

The position of the mutations (Table 1 and Fig. 3) are expressed in both c.DNA and g.DNA. For cDNA numbering, + 1 corresponds to the A of the ATG translation initiation codon in the reference sequence. cDNA variations in nucleotides are in accordance with GenBank entry NM_017671.4, while g.DNA positions were calculated from those using the position converter available in the web-based software Mutalyzer (build hg38, www.mutalyzer.nl) [56]. If only one mutation is mentioned, then it is in a homozygous state. The mapping along protein domains was not performed because most of the mutations are stop codons and likely to degrade the mRNA.

### Statistical analysis

Cumulative risk was obtained by calculating the number of new cases over the population at risk for each age segment. To compare the distribution of the mutations along the FERMT1 gene, parametric and non-parametric tests (Chi square, q-q test, ANOVA and Kruskal-Wallis test) were performed using R package v.3.1.2. Differences between groups were considered significant at *P* < 0.05.

## Data Availability

The datasets used and/or analysed during the current study are available from the corresponding author on reasonable request.
